# Melatonin Increases Fetal Weight in Wild-Type Mice but Not in Mouse Models of Fetal Growth Restriction

**DOI:** 10.3389/fphys.2018.01141

**Published:** 2018-08-15

**Authors:** Lewis J. Renshall, Hannah L. Morgan, Hymke Moens, David Cansfield, Sarah L. Finn-Sell, Teresa Tropea, Elizabeth C. Cottrell, Susan Greenwood, Colin P. Sibley, Mark Wareing, Mark R. Dilworth

**Affiliations:** ^1^Maternal and Fetal Health Research Centre, Division of Developmental Biology and Medicine, Faculty of Biology, Medicine and Health, University of Manchester, Manchester, United Kingdom; ^2^Manchester Academic Health Science Centre, Manchester University NHS Foundation Trust, St. Mary’s Hospital, Manchester, United Kingdom

**Keywords:** FGR, IUGR, melatonin, pregnancy, mouse models, eNOS

## Abstract

Fetal growth restriction (FGR) presents with an increased risk of stillbirth and childhood and adulthood morbidity. Melatonin, a neurohormone and antioxidant, has been suggested as having therapeutic benefit in FGR. We tested the hypothesis that melatonin would increase fetal growth in two mouse models of FGR which together represent a spectrum of the placental phenotypes in this complication: namely the endothelial nitric oxide synthase knockout mouse (eNOS^-/-^) which presents with abnormal uteroplacental blood flow, and the placental specific *Igf2* knockout mouse (P0^+/-^) which demonstrates aberrant placental morphology akin to human FGR. Melatonin (5 μg/ml) was administered via drinking water from embryonic day (E)12.5 in C57Bl/6J wild-type (WT), eNOS^-/-^, and P0^+/-^ mice. Melatonin supplementation significantly increased fetal weight in WT, but not eNOS^-/-^ or P0^+/-^ mice at E18.5. Melatonin did, however, significantly increase abdominal circumference in P0^+/-^ mice. Melatonin had no effect on placental weight in any group. Uterine arteries from eNOS^-/-^ mice demonstrated aberrant function compared with WT but melatonin treatment did not affect uterine artery vascular reactivity in either of these genotypes. Umbilical arteries from melatonin treated P0^+/-^ mice demonstrated increased relaxation in response to the nitric oxide donor SNP compared with control. The increased fetal weight in WT mice and abdominal circumference in P0^+/-^, together with the lack of any effect in eNOS^-/-^, suggest that the presence of eNOS is required for the growth promoting effects of melatonin. This study supports further work on the possibility of melatonin as a treatment for FGR.

## Introduction

Fetal growth restriction (FGR), the inability of a fetus to achieve its genetic growth potential, affects between 5 and 10% of pregnancies and is a major risk factor for stillbirth (Miller et al., 2008). In addition, FGR infants that survive are at higher risk of childhood morbidities such as cerebral palsy (Jarvis et al., 2003). There are also well-established correlations between being born small and an increased risk of a host of adulthood diseases including hypertension, diabetes, and stroke (Barker and Osmond, 1988; Barker et al., 1990; Osmond et al., 2007). Whilst there are many pathologies underlying FGR, the majority of cases are due to placental dysfunction (Baschat et al., 2007).

Despite these significant antenatal and postnatal consequences of FGR, there is no treatment. Current clinical management results in early delivery of the baby which is itself associated with poor fetal and neonatal outcomes (Bernstein et al., 2000; Resnik, 2002). Thus, the need for an effective treatment for FGR remains paramount. This lack of therapies for FGR is compounded by the fact that there is a reluctance to design drugs specifically for obstetric conditions (Fisk and Atun, 2008). Thus, there has been a drive for the re-purposing of therapeutics, licensed for use in other clinical diseases, which may be of translational benefit to obstetric medicine.

In order for potential treatments for FGR to progress toward clinical trials there is a need for well-characterized *in vivo* models of FGR in which to carry out pre-clinical efficacy testing. Two such models of FGR are the endothelial nitric oxide synthase knockout (eNOS^-/-^) mouse and the placental specific insulin-like growth factor 2 knockout mouse (P0^+/-^), which have been extensively characterized by ourselves and others (Shesely et al., 1996; Constancia et al., 2002; Dilworth et al., 2010, 2011; Kusinski et al., 2011, 2012; Kulandavelu et al., 2012, 2013; Stanley et al., 2012a). eNOS catalyses the conversion of L-arginine to nitric oxide (NO), a potent vasodilator that acts via smooth muscle cells (Moncada et al., 1991). Deletion of eNOS in mice (eNOS^-/-^) results in hypertension, also maintained during pregnancy (Shesely et al., 1996; Kusinski et al., 2012), and growth restricted fetuses that are 10–15% smaller than wild-type (WT) fetuses near term. In common with some cases of human FGR, uterine and umbilical artery blood flow velocity is reduced in eNOS^-/-^ mice compared with WT (Toal et al., 2008; Ghosh and Gudmundsson, 2009; McCowan et al., 2010) whilst uterine arteries of eNOS^-/-^ demonstrate reduced endothelium-dependent vasodilatation compared with WT (Kusinski et al., 2012; Stanley et al., 2012a). Placentas from eNOS^-/-^ mice exhibit oxidative stress (Stanley et al., 2012a) and reduced system A amino acid transport (Kusinski et al., 2012), akin to human FGR (Glazier et al., 1996; Biri et al., 2007; Shibata et al., 2008; Mert et al., 2012).

The P0^+/-^ mouse, in which a placental specific promoter of *Igf2* is deleted, is a model of late-onset FGR, with fetuses approximately 20% smaller than WT near term (Constancia et al., 2002; Dilworth et al., 2010, 2011). Placentas of P0^+/-^ mice also demonstrate a reduction in weight versus WT and this reduction precedes the onset of FGR (Constancia et al., 2002). P0^+/-^ placentas demonstrate aberrant placental morphology, in common with human FGR, with reduced surface area and increased thickness of the interhemal membrane, both of which contribute to a reduction in permeability to hydrophilic solutes (Mayhew et al., 2003; Sibley et al., 2004). Placental nutrient transfer is also aberrant in P0^+/-^ mice (Constancia et al., 2002, 2005; Dilworth et al., 2010). We have previously demonstrated that uterine and umbilical artery blood flow velocity in P0 mice^+/-^ is indistinguishable from WT mice (Dilworth et al., 2013). Together, eNOS^-/-^ and P0^+/-^ mice represent abnormal blood flow and defective placental transport phenotypes, respectively, that underlie placental dysfunction in FGR, thus providing suitable models in which to test potential therapeutics.

One such candidate therapeutic is melatonin, a naturally synthesized neurohormone primarily produced by the pineal gland and important in the establishment of circadian rhythms. Melatonin is a powerful antioxidant and in a rat model of FGR, in which dams were undernourished, maternal melatonin treatment normalized birth weights of growth restricted pups (Richter et al., 2009) with associated increases in the level of antioxidant enzymes including catalase and manganese-superoxide dismutase (SOD2) within the placenta. In a separate study, where FGR was induced by lipopolysaccharide (LPS) administration in mice, melatonin normalized fetal weights via reductions in placental oxidative stress and hypoxia (Chen et al., 2006). In pregnant sheep, intravenous infusion of melatonin increased umbilical artery blood flow via NO dependent mechanisms (Thakor et al., 2010).

eNOS^-/-^ and P0^+/-^ knockout mice provide two models that map onto differing phenotypes of placental dysfunction. The use of eNOS^-/-^ mice is akin to those FGR cases that present with abnormal uterine artery blood flow and endothelial dysfunction. The choice of this model also allows a discrete assessment of the importance of eNOS, via its deletion, in the effectiveness of melatonin in increasing fetal growth. The use of the P0^+/-^ mouse enables an assessment of melatonin in a model of late-onset FGR akin to women that do not present with abnormalities in uteroplacental blood flow but demonstrate placental pathology as evidenced by abnormal morphology and nutrient transfer. We thus tested the hypothesis that antenatal melatonin supplementation would increase fetal growth in the eNOS^-/-^ and P0^+/-^ mouse models of FGR, each representing a different placental pathology associated with FGR.

## Materials and Methods

### Animals

This study was carried out in accordance with the recommendations of the UK Animals (Scientific Procedures) Act of 1986 under Home Office licenses PPL 40/3385 and P9755892D. The protocols were approved by the Local Animal Welfare and Ethical Review Board (AWERB) of the University of Manchester.

Endothelial NO synthase knockout mice (eNOS^-/-^), stock number 002684, were purchased from Jackson Laboratories (Bar Harbor, ME, United States). C57Bl/6J mice (Envigo, United Kingdom), the background strain for eNOS^-/-^, were used as control mice (wild-type, WT) for the eNOS^-/-^ studies. Placental specific insulin-like growth factor 2 knockout mice (P0^+/-^) were a kind gift from Wolf Reik and Miguel Constancia (University of Cambridge) (Constancia et al., 2000). eNOS^-/-^ female mice were mated with eNOS^-/-^ male mice. WT female mice were mated with WT males. For P0 matings, P0^+/-^ male mice were mated with C57Bl/6J female mice which resulted in mixed litters of P0^+/-^ and WT (P0^+/+^, control) fetuses. All female mice were 10–16 weeks old at time of mating; male mice were 12–26 weeks old. Embryonic day 0.5 of gestation (E0.5) was determined by the discovery of a copulation plug (term = E19.5). All animals were provided with nesting material and housed in individually ventilated cages maintained under a constant 12 h light/dark cycle at 21–23°C with free access to food (BK001 diet, Special Dietary Services, United Kingdom) and water (Hydropac, Lab products Inc, Seaford, DE, United States).

On E12.5, animals were randomly assigned, using an online blocked randomization tool, into either control or treated groups. Researchers were not blinded to treatment group. Both treated and control groups were assessed concurrently. Those mice in the treated group were given 5 μg/ml melatonin (Sigma-Aldrich, United Kingdom) via drinking water. This dose was chosen according to another rodent study showing beneficial effects on fetal/birth weight (Richter et al., 2009). This study resulted in plasma concentrations of melatonin equivalent to those following the use of melatonin for jet lag (Herxheimer and Petrie, 2002) and similar to the proposed dose for the phase 1 pilot clinical trial in an FGR cohort (Alers et al., 2013). Due to poor solubility in water, melatonin was initially dissolved in 100% ethanol. The final concentration of ethanol in the drinking water was 0.05%. Mice were dosed until E18.5 with a fresh bottle made up at E15.5. Control animals had access to 0.05% ethanol in standard drinking water (vehicle). All dosing, including bottle changes, and harvesting of tissue took place in the morning between 8 am and 12 pm.

In total, 21 WT and 18 eNOS^-/-^ mice were placed on vehicle, and 21 WT and 20 eNOS^-/-^ mice were placed on melatonin treatment. For P0^+/-^ studies, 13 mice were placed on vehicle and 14 mice placed on melatonin. P0^+/-^ mice produce mixed litters of P0^+/-^ and WT pups (referred to as P0^+/+^ to distinguish from WT controls used as a comparison for eNOS^-/-^ mice). All mice were humanely euthanased at E18.5. Fetal and placental weights were recorded from all litters following weighing on a Mettler AC100 analytical balance (Mettler-Toledo, Leicester, United Kingdom). Fetal biometric measurements (crown:rump length, abdominal circumference and head circumference) were taken as previously described from 18 WT and 13 eNOS^-/-^ mice placed on vehicle, and 20 WT and 17 eNOS^-/-^ mice placed on melatonin treatment. For P0^+/-^ studies, 10 litters on vehicle and 11 litters on melatonin had biometric measurements taken (Kusinski et al., 2012).

### Fetal Weight Frequency Distribution Curves

Fetal weight histograms were constructed as previously described (Dilworth et al., 2011) and a non-linear regression performed (Gaussian distribution). The 5th and 10th percentile weights of the WT vehicle group were calculated as:

(-Z critical value × SD) + mean

where Z critical value = 1.645 (5th centile) or 1.2816 (10th centile) and SD = standard deviation.

### Wire Myography

#### Uterine Arteries

Main loop uterine arteries were dissected, at E18.5, from eNOS^-/-^ (five vehicle and five melatonin treated) and WT mice (eight vehicle and eight melatonin treated). For P0^+/-^ studies there were only two groups (nine vehicle and seven melatonin treated) since P0^+/+^ and P0^+/-^ fetuses are littermates and thus ultimately supplied by the same main uterine artery loop *in utero*. Wire myography was performed as described previously (Kusinski et al., 2009). Uterine artery diameters were estimated following normalization to an internal diameter of 0.9 of *L*_13.3_ kPa (where *L* = internal circumference the vessel would have if unmounted and subjected to a transmural pressure of 100 mm Hg). Constriction was measured using phenylephrine (PE; 10^-5^ M) and expressed as a percentage of the maximum constriction to a high potassium salt solution (KPSS, 120 mM). In uterine arteries pre-constricted with PE, endothelium-dependent relaxation was assessed by exposure to acetylcholine (ACh; 10^-10^ M to 10^-5^ M).

#### Umbilical Arteries

Umbilical arteries were dissected at E18.5 from WT and eNOS^-/-^ mice (from five litters placed on vehicle and five litters treated with melatonin) and from P0^+/+^ and P0^+/-^ mice (seven litters placed on vehicle and seven litters treated with melatonin). Pups, from which corresponding umbilical arteries were dissected, were selected at random. Wire myography was performed as described previously (Kusinski et al., 2009). Umbilical artery diameters were estimated following normalization to an internal diameter of 0.9 of *L*_5.1_ kPa. Dose-dependent constriction to 10^-10^ to 2 × 10^-6^ M U46619 was assessed and expressed as a percentage of the maximum constriction to KPSS (120 mM). Dose dependent relaxation to 10^-10^ to 10^-5^ M sodium nitroprusside (SNP) was assessed and calculated as a percentage of EC_80_ U46619 constriction.

### Data and Statistical Analyses

Sample sizes were determined using Altman’s nomogram based upon a 5% significance level and 80% statistical power, with the unit of analysis being number of litters. Fetal weight was the primary outcome and studies were powered according to data from similar studies by our group (Dilworth et al., 2013). All data were normally distributed following a D’Agostino and Pearson normality test and are shown as mean ± SEM. A Generalized Linear Mixed Models approach, with each litter (dam) used as a random effect, was used to assess whether there was a significant effect of genotype and/or treatment for fetal/placental weights and biometric measures. A Sequential Sidak multiple comparisons test was then used to test for differences between groups. For uterine and umbilical artery wire myography, a two-way ANOVA was used to assess the effects of genotype and treatment, followed by Bonferroni’s post-test to compare differences between individual groups. *P* < 0.05 was deemed statistically significant. Statistical analyses were performed using either IBM SPSS Statistics v21.0 (IBM, New York, NY, United States) or Graphpad prism v6.0 (Graphpad, La Jolla, CA, United States).

## Results

### Fluid Consumption

Following commencement of melatonin supplementation, amount of water drunk (with either melatonin or vehicle added) was measured until E18.5. There was no difference in amount of fluid drunk (ml/day) according to either genotype or treatment in eNOS^-/-^ and WT mice (WT vehicle 7.6 ± 0.4, WT melatonin 6.6 ± 0.2, eNOS^-/-^ vehicle 7.2 ± 0.5, eNOS^-/-^ melatonin 6.8 ± 0.4, two-way ANOVA). There was no difference in amount of fluid drunk between treatment groups in dams carrying P0^+/+^ and P0^+/-^ (6.7 ± 0.3 vehicle group versus 7.1 ± 0.5 in the melatonin treated group, Mann–Whitney test).

### Fetal and Placental Weights and Fetal Biometric Measurements

#### eNOS^-/-^ Mice

**FIGURE 1:**
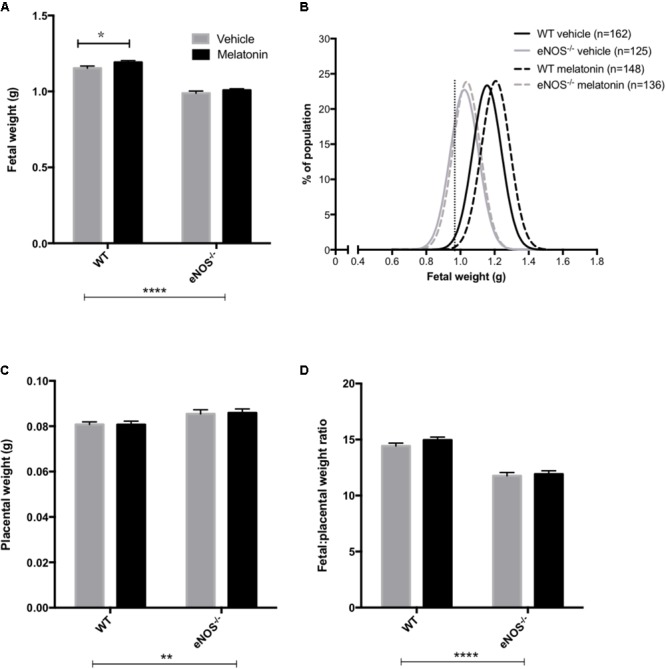
Fetal and placental weights and fetal:placental weight ratio in WT and eNOS^-/-^ mice at embryonic day 18.5. In **(A,C,D)**, mean + SEM is shown. **(B)** is a % frequency distribution plot of individual fetal weights with vertical dotted lines denoting the 5th centile (0.96 g, line on left) and 10th centile (1.06 g, line on right) of WT vehicle weights and *n* = number of fetuses. Number of litters are as follows; WT vehicle *N* = 21, WT melatonin *N* = 21, eNOS^-/-^ vehicle *N* = 18, eNOS^-/-^ melatonin *N* = 20. ^∗^*P* < 0.05, ^∗∗^*P* < 0.01, ^∗∗∗∗^*P* < 0.0001. Generalized linear mixed models analysis with sequential Sidak post-test.

Litter sizes (mean ± SEM) were as follows; WT vehicle 7.1 ± 0.4, WT melatonin 7.0 ± 0.4, eNOS^-/-^ vehicle 6.9 ± 0.4, eNOS^-/-^ melatonin 7.0 ± 0.4. Litter size was not significantly affected by either genotype or melatonin treatment. Fetal and placental weights are shown in **Figure 1**. Fetal weight (**Figure 1A**) was significantly lower in eNOS^-/-^ versus WT, independent of treatment (*P* < 0.001). Additionally, melatonin significantly increased fetal weight in WT mice (mean fetal weight 1.15 ± 0.02 g in WT vehicle group versus 1.20 ± 0.01 g in WT melatonin, *P* < 0.05) but not in eNOS^-/-^ mice (0.99 ± 0.02 g in eNOS^-/-^ versus 1.01 ± 0.01 g in eNOS^-/-^ melatonin). To explore this effect on fetal weight in further detail, fetal weight distribution curves (**Figure 1B**) were constructed. The curves for eNOS^-/-^ mice treated with vehicle and melatonin were similar, but the curve for WT mice treated with melatonin was shifted to the right of the curve for WT mice receiving vehicle. In this WT vehicle treated group, 0.968 g and 1.007 g were representative of the 5th and 10th fetal weight centiles, respectively. When assessing the percentage of fetuses with weights falling below the 5th centile of WT vehicle treated mice, it was observed that 2% of WT melatonin treated, 30% of eNOS^-/-^ vehicle and 30% of eNOS^-/-^ melatonin treated fetuses fell below this 5th weight centile. When assessing the percentage of fetuses weighing below the 10th centile of WT mice treated with vehicle, typically defined as the small for gestational age threshold in humans, there was a statistically significant reduction in the percentage of melatonin treated fetuses (3%) below this 10th centile value compared with WT vehicle (*P* < 0.01, chi-squared test). There was no difference in the percentage of fetuses weighing below the 10th centile of the WT vehicle treated population between eNOS^-/-^ mice receiving melatonin versus eNOS^-/-^ treated with vehicle (45% versus 47%, respectively). Placental weights (**Figure 1C**) were significantly elevated in eNOS^-/-^ versus WT mice, independent of treatment (*P* < 0.01). Melatonin treatment had no significant effect on placental weight in either eNOS^-/-^ or WT mice. The fetal:placental weight ratio (**Figure 1D**) was significantly reduced in eNOS^-/-^ mice independent of treatment and was unaffected by melatonin treatment in both WT and eNOS^-/-^ mice. Fetal biometric measurements are shown in **Table 1**. Fetal crown:rump length, abdominal circumference and head circumference were all significantly reduced in eNOS^-/-^ versus WT mice, independent of treatment (*P* < 0.001); melatonin treatment did not alter crown:rump length or abdominal circumference in either WT or eNOS^-/-^ fetuses but head circumference was increased following melatonin treatment in WT fetuses only (*P* < 0.01).

**Table 1:** Fetal biometric measures at E18.5 in WT and eNOS^-/-^ mice.

	WT vehicle	WT melatonin	eNOS^-/-^ vehicle	eNOS^-/-^ melatonin
Crown:rump length (mm)	28.2 ± 0.5	28.4 ± 0.5	26.6 ± 0.7^a,b^	26.6 ± 0.6^a,b^
Abdominal circumference (mm)	25.8 ± 0.3	26.0 ± 0.3	24.0 ± 0.5^a,b^	23.9 ± 0.3^a,b^
Head circumference (mm)	25.0 ± 0.2	24.7 ± 0.3	24.4 ± 0.4^a,b^	24.2 ± 0.3^a,b^

*Number of litters as follows; WT vehicle *N* = 18, WT melatonin *N* = 20, eNOS^-/-^ vehicle *N* = 13, eNOS^-/-^ melatonin *N* = 17. ^a^*P* < 0.001 compared with WT vehicle, ^b^*P* < 0.001 compared with WT melatonin, generalized linear mixed models with sequential Sidak post-test.*

#### P0^+/-^ Mice

**FIGURE 2:**
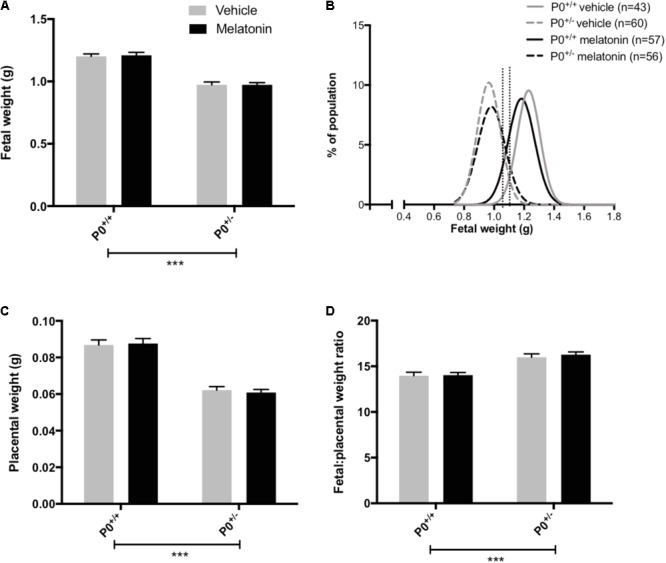
Fetal and placental weights in P0^+/+^ and P0^+/-^ mice at embryonic day 18.5. In **(A,C,D)**, mean + SEM is shown. **(B)** is a % frequency distribution plot of individual fetal weights with vertical dotted lines denoting the 5th centile (1.02 g, left hand line) and 10th centile (1.06 g, right hand line) of P0^+/+^ vehicle weights and *n* = number of fetuses. Number of litters are as follows; P0^+/+^ vehicle *N* = 13, P0^+/+^ melatonin *N* = 14, P0^+/-^ vehicle *N* = 13, P0^+/-^ melatonin *N* = 14. ^∗∗∗^*P* < 0.001 Generalized linear mixed models analysis with sequential Sidak post-test.

Litter size (mean ± SEM) was comparable between vehicle (8.0 ± 0.3) and melatonin treated (8.0 ± 0.4) mice. Fetal and placental weights are presented in **Figure 2**. Fetal weight was significantly reduced in P0^+/-^ mice compared with P0^+/+^ controls (*P* < 0.0001) with this effect being independent of treatment group (**Figure 2A**). Melatonin supplementation had no significant effect on fetal weight in either genotype. Fetal weight distribution curves appeared comparable between P0^+/-^ treated and untreated groups (**Figure 2B**) but there was a suggestion that melatonin supplementation had shifted the P0^+/+^ curve to the left. In order to explore this further, we assessed the number of fetuses falling below the 5th and 10th centile of P0^+/+^ fetal weights. Values for the 5th and 10th centiles were 1.02 g and 1.06 g, respectively, with 4% and 7% of the P0^+/+^ melatonin population falling below these values, not significantly different from the P0^+/+^ controls. Placental weights were significantly reduced in P0^+/-^ versus P0^+/+^ mice (*P* < 0.0001, **Figure 2C**) and this effect was independent of treatment group. Melatonin supplementation did not alter placental weight in either P0^+/+^ or P0^+/-^ mice. Fetal:placental weight ratio (**Figure 2D**) was significantly increased in P0^+/-^ mice independent of treatment group (*P* < 0.0001) but melatonin supplementation did not alter F:P ratio in either P0^+/+^ or P0^+/-^ mice. Crown:rump lengths, abdominal and head circumferences are shown in **Table 2**. There was no effect of melatonin treatment on crown-rump length in P0^+/+^ fetuses. Crown:rump length in P0^+/-^ fetuses supplemented with melatonin *in utero* also failed to reach statistical significance (*P* = 0.06). Melatonin supplementation increased abdominal circumference in P0^+/-^ mice (*P* < 0.05) but there was no effect on P0^+/+^ fetuses. Genotype, independent of treatment, had no significant effect on head circumference; melatonin supplementation did not alter head circumference in either P0^+/+^ or P0^+/-^ fetuses.

**Table 2:** Fetal biometric measures at E18.5 in P0^+/+^ and P0^+/-^ mice.

	P0^+/+^ vehicle	P0^+/+^ melatonin	P0^+/-^ vehicle	P0^+/-^ melatonin
Crown:rump length (mm)	28.8 ± 0.3	28.6 ± 0.2	27.4 ± 0.3^a,b^	26.7 ± 0.3^a,b^
Abdominal circumference (mm)	26.4 ± 0.5	26.9 ± 0.4	23.6 ± 0.4^a,b^	24.6 ± 0.3^a,b,c^
Head circumference (mm)	25.1 ± 0.4	25.1 ± 0.1	24.8 ± 0.3	24.9 ± 0.2

*Number of litters as follows; P0^+/+^ vehicle *N* = 10, P0^+/+^ melatonin *N* = 11, P0^+/-^ vehicle *N* = 10, P0^+/-^ melatonin *N* = 11. ^a^*P* < 0.001 compared with P0^+/+^ vehicle, ^b^*P* < 0.001 compared with P0^+/+^ melatonin, ^c^*P* < 0.05 compared with P0^+/-^ vehicle, generalized linear mixed models with sequential Sidak post-test.*

### *Ex vivo* Uterine Artery Function

#### eNOS^-/-^ Mice

**FIGURE 3:**
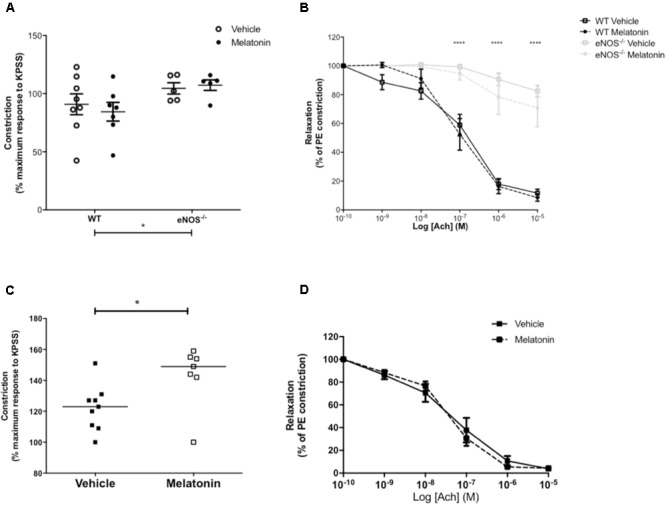
Constriction and relaxation responses of uterine arteries from WT and eNOS^-/-^ mice **(A,B)** and P0^+/+^ and P0^+/-^ mice **(C,D)** at embryonic day 18.5. **(A,C)** Constriction to 10^-5^ M phenylephrine shown as a percentage of the KPSS constriction; each dot represents a single animal, line denotes mean. **(B,D)** Dose dependent relaxation to acetylcholine is shown as a percentage of constriction to phenylephrine. Data are presented as mean ± SEM **(A,B,D)** or all data points with line denoting median **(C)**. Number of mice were as follows; WT *N* = 8, eNOS^-/-^ *N* = 5, for both vehicle and melatonin groups. For uterine arteries from P0 matings, *N* = 9 for vehicle and *N* = 7 for melatonin treated. Statistical analysis in **(A,B)** was carried out by two-way ANOVA to assess the effect of genotype and treatment with Bonferroni post-test to compare individual groups. In **(C)**, a Mann–Whitney test was performed and in **(D)** a two-way ANOVA to assess the effect of treatment and [Ach]. ^∗^*P* < 0.05, ^∗∗∗∗^*P* < 0.0001 for effect of genotype independent of treatment.

Uterine artery diameters (μM, mean ± SEM) were as follows; WT vehicle 211 ± 17, WT melatonin 217 ± 25, eNOS^-/-^ vehicle 147 ± 13, eNOS^-/-^ melatonin 182 ± 25. Diameters were reduced in eNOS^-/-^ versus WT, independent of treatment group (*P* < 0.05, two-way ANOVA). There was no difference in uterine artery diameter following melatonin treatment in either WT or eNOS^-/-^ mice. Maximal constriction to 10^-5^ M PE was greater in uterine arteries of eNOS^-/-^ versus WT mice, independent of treatment group (*P* < 0.05). Melatonin treatment had no effect on constriction in either WT or eNOS^-/-^ mice (**Figure 3A**). ACh caused significant relaxation of uterine arteries from WT mice following pre-constriction with PE; this relaxation was significantly blunted in arteries of eNOS^-/-^ mice independent of treatment (*P* < 0.0001). Melatonin had no significant effect on uterine artery relaxation to Ach in either WT or eNOS^-/-^ mice (**Figure 3B**).

#### P0^+/-^ Mice

Uterine artery diameters (μM, mean ± SEM) were not significantly different between vehicle (253 ± 19) and melatonin treated dams (238 ± 11). Uterine arteries from melatonin treated mice demonstrated greater maximal constriction to PE compared with the vehicle group (*P* < 0.05, **Figure 3C**). There was no significant difference in relaxation of uterine arteries to Ach (as % of constriction to PE) between vehicle and melatonin treated mice (**Figure 3D**).

### *Ex vivo* Umbilical Artery Function

#### eNOS^-/-^ Mice

**FIGURE 4:**
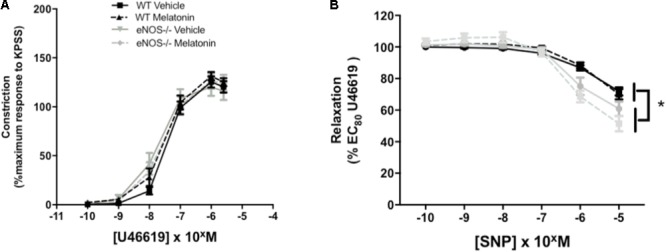
Constriction and relaxation responses of umbilical arteries from WT and eNOS^-/-^ mice at embryonic day 18.5. **(A)** Dose-dependent constriction to 10^-10^ to 2 × 10^-6^ M U46619 shown as a percentage of the maximum constriction to KPSS. **(B)** Dose dependent relaxation to sodium nitroprusside (SNP) is shown as a percentage of EC_80_ U46619 constriction. Data are presented as mean ± SEM. N’s as follows (*N* = litters, *n* = pups); WT vehicle *N* = 5, *n* = 20, WT melatonin *N* = 5, *n* = 17 eNOS^-/-^ vehicle *N* = 5, *n* = 12, eNOS^-/-^ melatonin *N* = 5, *n* = 17. Statistical analysis was carried out by two-way ANOVA to assess the effect of genotype and treatment with Bonferroni post-test to compare individual groups. ^∗^*P* < 0.05 WT vs. eNOS^-/-^.

Umbilical artery diameters (μM, mean ± SEM) were as follows; WT vehicle 474 ± 26, WT melatonin 455 ± 38, eNOS^-/-^ vehicle 468 ± 25, eNOS^-/-^ melatonin 416 ± 17. There was no significant effect of genotype or treatment on umbilical artery diameter. There was no effect of genotype or treatment on constriction of umbilical arteries to U46619 (**Figure 4A**). Relaxation of umbilical arteries to SNP (as % EC_80_ U46619 preconstriction) was genotype-dependent with eNOS^-/-^ mice, independent of treatment group, showing increased relaxation to SNP versus WT (*P* < 0.05). Melatonin treatment had no effect on umbilical artery relaxation to SNP (**Figure 4B**).

#### P0^+/-^ Mice

**FIGURE 5:**
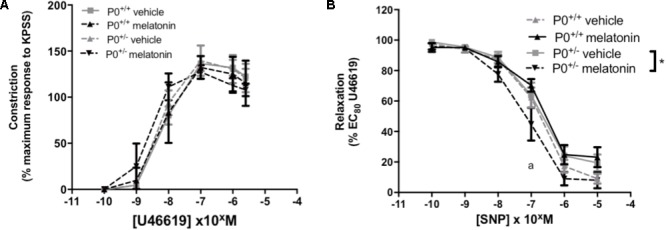
Constriction and relaxation responses of umbilical arteries from P0^+/+^ and P0^+/-^ mice at embryonic day 18.5. **(A)** Dose-dependent constriction to 10^-10^ to 2 × 10^-6^ M U46619 shown as a percentage of the maximum constriction to KPSS. **(B)** Dose dependent relaxation to sodium nitroprusside (SNP) is shown as a percentage of EC_80_ U46619 constriction. Data are presented as mean ± SEM. N’s as follows (*N* = litters, *n* = pups); P0^+/+^ vehicle *N* = 7, *n* = 12, P0^+/+^ melatonin *N* = 7, *n* = 12, P0^+/-^ vehicle *N* = 7, *n* = 12, P0^+/-^ melatonin *N* = 7, *n* = 9. Statistical analysis was carried out by two-way ANOVA to assess the effect of genotype and treatment with Bonferroni post-test to compare individual groups. ^∗^*P* < 0.05. ^a^*P* < 0.05 P0^+/-^ melatonin vs. P0^+/+^ vehicle and P0^+/+^ melatonin at 10^-7^ M.

Umbilical artery diameters (μM, mean ± SEM) were as follows; P0^+/+^ vehicle 499 ± 31, P0^+/+^ melatonin 492 ± 36, P0^+/-^ vehicle 499 ± 21, P0^+/-^ melatonin 470 ± 32. There was no significant effect of genotype or treatment on umbilical artery diameter. Constriction of umbilical arteries to U46619 was not significantly different between any groups (**Figure 5A**). Relaxation of umbilical arteries to SNP (expressed as a % EC_80_ U46619 preconstriction, **Figure 5B**) was not significantly affected by genotype alone but there was a treatment effect. *Post hoc* tests revealed that umbilical arteries from P0^+/-^ melatonin mice demonstrated significantly increased relaxation to SNP than P0^+/-^ treated by vehicle alone. Additionally, P0^+/-^ mice showed greater relaxation to SNP compared with P0^+/+^ mice in the vehicle and melatonin groups, though this effect was limited to an SNP dose of 10^-7^ M only.

## Discussion

Contrary to our hypothesis, melatonin failed to increase fetal weight in eNOS^-/-^ or P0^+/-^ mice, two mouse models of FGR which demonstrate different underlying placental pathology, but did increase fetal weight in WT mice. Melatonin supplementation *in utero* did, however, result in increased fetal abdominal circumference in P0^+/-^ mice. Despite the lack of a vascular phenotype in P0^+/-^ mice, melatonin supplementation increased relaxation of P0^+/-^ umbilical arteries to the NO-donor sodium nitroprusside. Although the mechanisms underpinning the increased fetal weight in WT mice and increased abdominal circumference in P0^+/-^ mice remain to be fully understood; the lack of any effect in eNOS^-/-^ mice suggest that the effects of melatonin are mediated, at least in part, via the presence of eNOS, suggestive of a potential role for NO. In the case of WT mice, melatonin has growth promoting effects even in the absence of a uterine or umbilical blood flow abnormality. Similarly, the data in P0^+/-^ mice suggests that melatonin may alter fetal growth, at least in terms of increased abdominal circumference, in cases of FGR in which there is no detectable uterine or umbilical blood flow abnormality. Consideration of these mechanisms is especially important as a phase 1 pilot clinical trial to assess the effectiveness of melatonin in pregnancies complicated by severe early onset FGR has commenced (Alers et al., 2013).

Fetal weights in untreated WT and eNOS^-/-^ mice were comparable with previous reports and confirmed the FGR phenotype of eNOS^-/-^ mice at E18.5 (Stanley et al., 2012a; Poudel et al., 2013) whilst the reduced eNOS^-/-^ fetal:placental weight ratio confirmed previous observations suggesting a reduced transport efficiency of the eNOS^-/-^ placenta (Kusinski et al., 2012; Stanley et al., 2012a; Poudel et al., 2013). Fetal and placental weights in P0^+/-^ mice were comparable to previous reports (Constancia et al., 2002; Dilworth et al., 2010, 2011). Previous studies have suggested that melatonin increased fetal weight in a mouse model of LPS-induced FGR (Chen et al., 2006) and FGR following single umbilical artery ligation in sheep (Tare et al., 2014). Additionally, in an undernourished rat model of FGR (Richter et al., 2009), melatonin increased birth weight but this effect was not apparent prior to delivery. In these studies, the effect of increased fetal/birth weight was observed in the FGR groups only, as opposed to the present study where the effect was limited to WT mice. As noted above, this may be explained by a requirement for the presence of eNOS in the mechanism underpinning this increased fetal growth. This is supported by studies showing that the production of NO via NOS enzymes is important in terms of melatonin’s actions of increasing umbilical blood flow in a sheep model of FGR (Thakor et al., 2010). It has also been demonstrated that, following single umbilical artery ligation in sheep, melatonin treatment *in utero* rescued endothelial-dependent coronary artery function in neonatal FGR lambs by increasing NO bioavailability (Tare et al., 2014). Additionally, melatonin alters fetal cardiometabolic responses in fetal lambs exposed to acute hypoxia *in utero*, including reductions in fetal arterial blood pressure. These effects were prevented via a NO blockade, providing further evidence of melatonin’s effects in terms of increasing NO bioavailability (Thakor et al., 2015).

The increased fetal growth seen here in appropriately grown WT fetuses following maternal melatonin supplementation has been observed before. In sheep, melatonin administered *in utero* increased fetal ponderal index and abdominal girth, with a trend toward increased fetal weight (Lemley et al., 2012), in lambs that were appropriately grown, with no effect on nutrient-restricted FGR lambs. This increased abdominal girth, or circumference, was noted in the present study in P0^+/-^ pups treated with melatonin but crown:rump length was unaffected (*P* = 0.06). Whilst umbilical blood flow velocity was not measured in the current study, the greater relaxation of umbilical arteries in melatonin treated P0^+/-^ mice is consistent with the increased abdominal circumference (Scorza et al., 1991; Stanley et al., 2012b). Studies investigating other potential therapies for FGR, including sildenafil citrate, have previously reported an increased fetal abdominal circumference growth velocity following treatment (von Dadelszen et al., 2011) and outlined the importance of serial abdominal circumference measures, via ultrasound, as a means of assessing growth velocity before and after treatment. Interestingly, given the increased fetal weight in WT mice following administration of melatonin, one may expect a similar increase in P0^+/+^ mice, the WT equivalent in a mixed P0 knockout mouse litter. However, melatonin did not alter P0^+/+^ fetal weight. Whilst the reasons for this can only be speculated upon at this time, it is apparent that P0^+/+^ mice treated with vehicle had a higher mean fetal weight (1.20 ± 0.02 g) than WT vehicle treated mice (1.15 ± 0.02 g) and a weight comparable to WT mice treated with melatonin (1.20 ± 0.02 g). This is presumably because on average, P0 knockout litters consist of 50% growth restricted P0^+/-^ fetuses and there will likely be a relative surplus of maternal nutrients available to enable maximal growth of P0^+/+^ pups. This phenomenon is similar to the inverse relationship described between litter size and fetal weight in late gestation in the mouse (Ishikawa et al., 2006) which demonstrates that maternal nutrient availability is a limiting factor for fetal growth. It has been reported that melatonin alters uteroplacental amino acid flux and increases fetal uptake of branched chain amino acids in a sheep model of FGR following maternal undernutrition (Lemley et al., 2013). Thus, future studies examining maternofetal amino acid transfer in melatonin treated WT mice may hint toward a potential mechanism for the increased fetal growth observed.

Melatonin treatment *in utero* increased relaxation of umbilical arteries of P0^+/-^ mice in response to SNP, a NO donor, demonstrating an increased sensitivity that is endothelial-independent. This gives further credence to the notion that NO is important in the actions of melatonin. A recent study in sheep fed a nutrient restricted diet, demonstrated that cotyledonary arteries (described as secondary branches of the umbilical artery) from those sheep on the restricted diet demonstrated increased sensitivity to SNP which was subsequently reversed by melatonin supplementation (Shukla et al., 2014). In the same study, ewes on a normal diet demonstrated that the opposite was true, i.e.; melatonin increased sensitivity to SNP in cotyledonary arteries. These latter data appear to fit with the P0 umbilical data presented here.

In a number of studies, there is direct evidence of melatonin’s antioxidant properties. In a study by Chen et al. (2006), melatonin supplementation reduced fetal mortality and increased fetal weight in a model of intrauterine death following LPS administration. This was suggested to be due to a significant reduction in LPS-induced lipid peroxidation and normalization of the LPS-induced decrease in placental glutathione (Chen et al., 2006; Wang et al., 2011). Richter et al. (2009) demonstrated that, in a rat model of induced FGR following maternal undernutrition, melatonin increased fetal weight with an accompanying increase in levels of placental catalase and manganese-superoxide dismutase (Mn-SOD, also known as SOD2). In the current study, only WT mice demonstrated an increase in fetal weight following melatonin supplementation. WT mice, and thus WT placentas, will not be subject to excess levels of oxidative stress and thus it is unlikely that the effect of melatonin on WT fetal growth is via antioxidant mechanisms. Thus, the mechanism underpinning this increased WT fetal weight remains elusive and future studies examining this present a logical next step.

Whilst the studies discussed above relate to a number of different species, with melatonin administered by a variety of routes (via drinking water, subcutaneous, i.v. infusion), there is a body of evidence that supports the fact that melatonin treatment *in utero* is able to increase fetal growth (Chen et al., 2006; Richter et al., 2009; Lemley et al., 2012; Tare et al., 2014). However, in the current study, melatonin failed to increase fetal weight in either of two mouse models of FGR each of which demonstrates different placental pathologies observed in cases of human FGR (McCowan et al., 1988; Mayhew et al., 2003; Toal et al., 2008; Ghosh and Gudmundsson, 2009). It is important to note that whilst melatonin did increase fetal weight in WT mice, this is not representative of the severe FGR cases that would be targeted clinically for intervention. The current study does, however, add weight to the importance of NO bioavailability in the actions of melatonin on fetal growth by targeting a mouse model with a specific deletion of eNOS. Delineating the pathways by which melatonin does act to increase fetal growth will be paramount, particularly as the ability to stratify cases of FGR improves (Audette and Kingdom, 2017; Gaccioli et al., 2017).

## Author Contributions

LR, HLM, HM, DC, SF-S, TT, and EC performed the research. SG and CS contributed to the conception and design of the work. MW and MD contributed to the conception and design of the work and performed the research. All authors were involved in drafting the paper.

## Conflict of Interest Statement

The authors declare that the research was conducted in the absence of any commercial or financial relationships that could be construed as a potential conflict of interest.
